# Effects of biofeedback-based sleep improvement program on urinary symptoms and sleep patterns of elderly Korean women with overactive bladder syndrome

**DOI:** 10.1186/s12894-019-0540-y

**Published:** 2019-11-08

**Authors:** Jooyeon Park, Choal Hee Park, Sang-Eun Jun, Eun-Ju Lee, Seung Wan Kang, Nahyun Kim

**Affiliations:** 1 College of Nursing, Keimyung University, Daegu, Republic of Korea; 2 Department of Urology, Keimyung University School of Medicine, Daegu, Republic of Korea; 3 College of Nursing, Seoul National University, Seoul, Republic of Korea

**Keywords:** Overactive bladder syndrome, Sleep, Autonomic nervous system

## Abstract

**Background:**

The prevalence of overactive bladder syndrome (OAB) increases with age. Sleep disturbances in elderly individuals with OAB is a common problem. The purpose of this study was to examine the effects of a biofeedback-based sleep improvement (BBSI) program on urinary symptoms and sleep patterns in elderly Korean women with OAB.

**Methods:**

A non-equivalent control group pre−/post-test design was used. Elderly women with OAB were assigned to an intervention group (*n* = 20) or a control group (*n* = 18). The BBSI program was implemented in the intervention group for 12 weeks, while two educational sessions of general sleep hygiene and lifestyle modification were provided to the control group. Using SPSS 23.0, the data were analyzed by descriptive analysis using the chi-square test, Fisher’s exact test, Mann-Whitney test, and Wilcoxon test.

**Results:**

After the 12-week BBSI program, significant improvements were found in the intervention group’s the square root of the mean squared differences of successive R-R intervals (*p* = 0.025), low frequency/high frequency ratio (*p* = 0.006), and epinephrine (*p* = 0.039). We also observed a significant difference in urinary symptoms, sleep efficiency, wake after sleep onset, number of awakenings, and number of awakenings within 3 h after sleep onset (*p* < 0.001, *p* = 0.004, *p* = 0.001, *p* = 0.001, and *p* = 0.048, respectively). However, no significant changes were found in these variables in the control group.

**Conclusions:**

The BBSI program effectively improved urinary symptoms and sleep patterns of elderly Korean women with OAB. Further longitudinal research is required to investigate the sustainability and effects of the BBSI program.

**Trial registration:**

KCT0003882. Date of registration: 02/05/2019. Retrospectively registered.

## Background

Sleep disorders in elderly individuals with overactive bladder syndrome (OAB) are a common problem. The prevalence of OAB in elderly individuals in the United States and Europe is 16.9 and 11.8%, respectively [1, 2]. OAB is characterized by major lower urinary tract dysfunction that causes urinary frequency, nocturia, and urinary urgency irrespective of urge incontinence [3]. Its prevalence increases with age [2] and lower physical activity [4] and is higher in females than in males [1]. Nocturia is the most uncomfortable lower urinary tract symptom in elderly individuals; it directly causes sleep disturbances and increases the risk of falls and fractures [5]. Frequent awakening due to symptoms of OAB disturbs the quality of sleep, resulting in severe fatigue in daytime activities [6].

The cause of OAB has not been identified yet; however, one or more physiological changes are thought to play a role in its development [7]. OAB without the presence of a neurological disease has been attributed to autonomic nervous system (ANS) abnormalities based on the role of the ANS in regulating bladder function [8]. The parasympathetic nervous system induces bladder contractions, while the sympathetic nervous system regulates the contractions of the bladder neck and urethral smooth muscle and relaxation of the bladder body [9]. Therefore, ANS imbalances can result in abnormal bladder activity [8].

Although elderly individuals can suffer from sleep disorders secondary to OAB, aging itself causes ANS changes, leading to increased and decreased activities of the sympathetic and parasympathetic nervous systems, respectively, thereby lowering the sleep quality [10]. Excessive sympathetic nervous system activity leads to frequent awakening and interferes with deep sleep, suggesting that sleep interventions in elderly women should be directed towards maintaining balance in the ANS [11, 12].

However, active efforts to solve such problems are lacking because most patients tend to perceive this issue as a natural phenomenon due to aging. Few studies have examined the management of symptoms in the elderly at the physiological level and few interventions are available to address sleep disorders. Sleep disorders are often considered characteristic of elderly patients. Additionally, there are limited drug therapies that can be used to improve sleep problems in elderly people with OAB. Anti-muscarinic drugs are the most commonly used ones in the treatment of OAB; however, long-term adherence to the management is very low due to the side effects, such as constipation and dry mouth [13, 14]. Particularly, elderly patients may be on multiple drugs for various comorbidities; therefore, care must be taken in prescribing these drugs. For this reason, several studies have suggested that multicomponent treatment should be considered as the first-line therapy for OAB in elderly people [15, 16].

In this study, we aimed to use mechanism-based interventions to improve sleep in elderly people with OAB and focused on using mechanisms to balance the ANS. A therapeutic approach towards balancing the ANS is essential to be an effective sleep intervention in elderly patients with OAB. The use of biofeedback from the sympathetic and parasympathetic nervous systems to balance the ANS appears to be an important approach in alleviating the symptoms of OAB. Additionally, biofeedback has been reported to be not only an effective intervention in patients with physical and psychological disorders secondary to ANS imbalances but also a suitable sleep intervention as well [17].

In the literature, changes in autonomic functions might be the underlying cause of OAB and lowered sleep quality. OAB also could be one of the major factors that interfere with deep sleep in the elderly. Therefore, we hypothesized that stabilizing the autonomic functions might reduce the symptoms of OAB and improve sleep.

## Methods

### Study design

This experimental study used a multi-site nonequivalent control group pre- and post-test design.

### Participants and procedures

This study was conducted between March and September 2018. Our study population was selected from the elderly women registered in two senior welfare centers in Korea. The inclusion criteria were the following: (1) age ≥ 65 years; (2) Overactive Bladder Symptom Score (OABSS) ≥ 3, and urgency score ≥ 2 [18]; (3) Pittsburgh Sleep Quality Index (PSQI) score ≥ 5 [19]; (4) Mini-Mental State Examination (MMSE) ≥ 24 [20]; and (5) no history of participation in other programs to improve sleep over the previous 6 months. Participants were excluded if they were consuming sleeping pills or OAB medications, had a history of urinary tract infection at the time of the survey, were diagnosed with neurological or psychiatric disorders or autonomic neuropathy, or were diagnosed with other diseases affecting autonomic function.

**Fig. 1:**
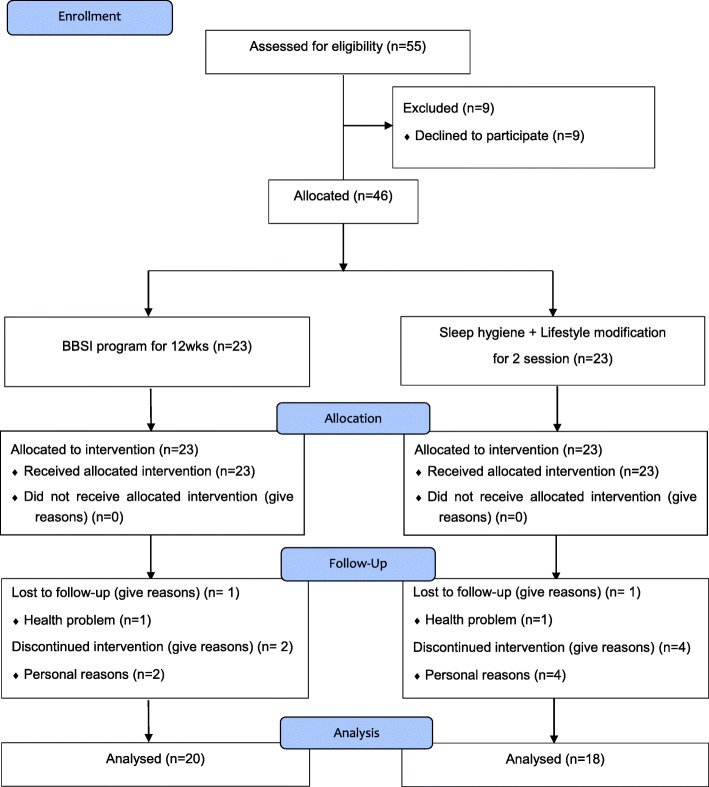
The CONSORT flow diagram. BBSI: Biofeedback based sleep improvement

To recruit the participants, we visited each senior welfare center and obtained permission to explain the study to prospective elderly participants. We then explained the study’s purpose and procedures and invited interested elderly patients to participate. We also used posters to invite elderly women to participate. After consulting the chief of each center, using a coin toss, one center was assigned to provide the experimental group and the other the control group. The intervention was initiated a week after the categorization. The flowchart of patient selection is depicted in Fig. 1. Of the 55 enrolled women, 9 women refused to participate in the study; therefore, 23 were included in the biofeedback-based sleep improvement (BBSI) program, while another 23 patients were only managed in terms of sleep hygiene and lifestyle modifications. Overall, eight patients were lost to follow-up and dropping out; the remaining 38 were followed up for 12 weeks (20 in the BBSI group vs. 18 in the control group).

For the pre-test, the participants arrived at the study location and rested for more than 30 min. Three research assistants administered the questionnaire and measured heart rate variability (HRV). For HRV measurements, the participants were suggested to relax and instructed to breathe comfortably without speaking. After the 12-week intervention, a follow-up evaluation was conducted with the same contents as those in the pre-test. The research assistants examined the same items as in the pre-test to minimize measurement errors.

### Sample size estimation

The sample size of this study was determined using the G power version 3.1.9 program. The sample size was calculated based on a two-sided test with significance level of 0.05, power of 0.80, and effect size of (d) 0.99 [21] in the study of sleep disorders. As a result, there were a total of 36 participants, 18 of whom were required in each group to evaluate differences between the two groups. Its actual power was 0.82. In the present study, 23 in the experimental group and 23 in the control group were chosen as candidates for participation in the program based on a dropout rate of 20%.

### Intervention

The intervention program was developed to improve sleep in elderly women with OAB. We designed the program with an emphasis on ANS balance, including individual intervention, group education, and telephone coaching. We prepared an intervention plan by reviewing the literature based on Overactive Bladder Syndrome Treatment Guidelines [22] and Sleep Hygiene Education provided by the Korean Society of Sleep Medicine [23]. The components of the program consisted of direct and indirect elements that have been previously reported to be effective for autonomic balance. Biofeedback was used for the direct approach for ANS balance [24], and lifestyle modifications, physical activity, and depression management—which were reported to be effective for ANS balance—were used as indirect approaches [25–27]. Additionally, behavioral therapy is very important in sleep disorders with OAB. Therefore, the participants were educated regarding sleep hygiene in consideration of OAB characteristics and pelvic floor muscle (PFM) contraction exercises were included in the program [28, 29]. The main outlines of the BBSI program were as follows: (1) individual intervention: HRV biofeedback training—a key component of ANS balance; (2) group training: behavioral therapy to reduce OAB symptoms and improve sleep, including lifestyle modifications, sleep hygiene, pelvic muscle contractions, physical activity, and depression management; and (3) telephone coaching: emotional support and individual goal setting. An additional file shows this in more detail [see Additional file 1].

The individual interventions consisted of weekly biofeedback training for 20 min for 12 weeks. For biofeedback training, a computerized biofeedback system (Procomp, Thought Technology, Montreal, QC, Canada) was used. By analyzing the changes in the visual heart rhythm and respiration, HRV biofeedback balances the ANS by controlling the respiratory rate. The intervention provider was an experienced urology nurse who was well-trained to provide biofeedback. Biofeedback confirmed the participant’s baseline status in pre-testing and maintained an ideal state (close to 1.5) of low frequency (LF) to high frequency (HF) ratio (LF/HF) during training [30].

Group education was conducted for 40 min once a week for 12 weeks. It consisted of sleep hygiene education, lifestyle modifications, PFM contraction exercises, physical activity, and depression management. The lifestyle modification program included body mass index control, caffeine restriction, and alcohol restriction. A pedometer was provided to increase each participant’s amount of physical activity. After each round of group training, individual counseling was provided to encourage each participant to achieve their goals.

Telephone coaching was conducted to encourage goal achievement and provide emotional support. We encouraged the use of biofeedback-based respiration techniques every day and set individual and daily goals to promote physical activity. We then confirmed that they practiced the same. Emotional support was provided after identifying the participants’ environmental and psychological statuses. Also, for positive reinforcement, the program provided participants with a workbook at the beginning of the program and rewarded participants who achieved the goal well during the mid-term check.

The control group received 40 min of training on sleep hygiene and lifestyle modifications in the 4th and 8th sessions of intervention, respectively. In order to maintain consistency in the contents of the two groups, education was conducted by the same educators in both groups. Additionally, before the program began, the researchers agreed on the materials to be provided to both groups and conducted the training in accordance with the agreed content. Sleep hygiene education consisted of the same 11 sleep hygiene items as those of the intervention group. Regarding lifestyle modifications, the control group was instructed to restrict caffeine and water intake, perform PFM exercises, and increase physical activity.

### Outcome measures

#### Measures of HRV

HRV was used to assess the sympathetic and parasympathetic activities since it enables qualitative, quantitative, and noninvasive analysis of global autonomic function [17, 31]. To assess ANS activity, HRV was measured with a heart rate monitoring device (Polar V800, Polar Electro Oy, Kempele, Finland). All R-R intervals collected through the wireless chest strap of the heart rate monitor were recorded with a 1000-Hz wireless heart rate monitor suitable for HRV analyses. After the measurement, the raw data were extracted by the Pro-trainer Polar 5 (version 5.40.171, Polar Electro) program and transferred to Kubios (version 2.0, 2008, Biosignal Analysis and Medical Imaging Group, Finland). We evaluated HRV with the converted values in Kubios.

The results of HRV demonstrate the values ​​in time and frequency domains for ANS activity. The parameters of the time domain analysis were the standard deviation of Normal to Normal R-R intervals (SDNN) and the square root of the mean squared differences of successive R-R intervals (RMSSD). SDNN estimates the overall HRV, while RMSSD represents parasympathetic activity. The parameters of the frequency domain analysis were total power (TP), LF, HF, and LF/HF ratio. TP reflects the control capacity of the ANS, LF (0.04–0.15 Hz) represents sympathetic activity, and HF (0.15–0.4 Hz) represents the parasympathetic activity. The LF/HF ratio represents the interaction and balance between the two systems [32].

With the participants breathing normally, we calculated SDNN, RMSSD, TP, LF, HR, and LF/HF ratio. The participants were instructed not to smoke or drink alcohol or caffeinated beverages after 10 pm on the night before data collection. Each participant was seated on a comfortable chair and the HRV monitor was placed on the left wrist. Participants were asked not to talk and breathe at a normal rate during the 5-min measurement.

#### Plasma catecholamine level

Epinephrine (epi) and norepinephrine were assayed using blood samples. Each participant was instructed to lie quietly for 30 min before the blood sampling. Venous blood (5 mL) was obtained in a heparin anticoagulation vacuum tube. Catecholamine levels were measured by high-performance liquid chromatography (Acclaim, Bio-Rad, United States of America).

#### OAB symptoms

OAB symptom severity was measured using 8 of the 33 items of the Overactive Bladder Questionnaire (OAB-q) developed by Cyone et al. [33]. The 8-item questionnaire consisted of 4 sub-areas: frequency, urgency, nocturia, and urge incontinence. Each item consists of a 6-point Likert scale ranging from “not affected at all” (1 point) to “strongly affected” (6 points). The symptom severity scale was calculated by the converted scores given by Coyne et al. [33] using a range of 0–100. The higher the score, the greater the severity of OAB symptoms. Cronbach’s α was 0.86 [33] vs. Cronbach’s α of 0.79 in our study.

#### Sleep patterns

We measured sleep efficiency (SE), wake after sleep onset (WASO), number of awakenings, and number of awakenings within 3 h of sleep onset using actigraphy (W-GT3X-BT, Actigraph, Inc., Pensacola FL, United States of America) to assess sleep. The actigraphy device can be worn on the waist or wrist using an elastic belt. In elderly individuals, wearing an actigraphy device on the wrist reportedly reduces the sensitivity of low-intensity activities when using walking aids [34, 35]; therefore, we chose to place it on the waist. The data collected by the actigraphy device was analyzed by Actilife 6 software to obtain SE, WASO, number of awakenings, and number of awakenings within 3 h of sleep onset. In this study, sleep was scored using medium threshold settings and default parameters for sleep scoring based on 1-min epochs. Since the measurement period of the actigraphy is at least 3 consecutive days [36], the participants wore the actigraphy device during sleep for 3 days. We also asked the participants to accurately collect information regarding bedtime and rise time for each night while they were wearing the actigraphy device.

### Data analysis

The data were analyzed using SPSS for Windows version 20.0 (SPSS Inc., Armonk, NY, United States of America). We used the Chi-square test, Fisher’s exact test, and Mann–Whitney test to test the homogeneity of the general characteristics of the experimental and control groups. The efficacy of the BBSI program was analyzed using the Wilcoxon test. Cronbach’s test was used to confirm reliability. *P* values < 0.05 were considered to be statistically significant.

## Results

Twenty elderly women with OAB treated with the BBSI program (BBSI group) and 18 with OAB without the BBSI program (control group) were included in the present study. The patients’ basic characteristics, clinical and laboratory parameters of ANS activity, OAB symptoms, and sleep patterns are summarized in Table 1. The mean age in the BBSI group and control group was 80.05 ± 4.21 and 81.06 ± 4.32 years, respectively. There were no intergroup differences in the educational level, marital status, monthly income, parity, caffeine intake, water intake, BMI, or OAB duration. Regarding the parameters of HRV, catecholamines, OAB symptoms, and sleep patterns did not differ between the groups.

**Table 1:** Homogeneity test for general characteristics and baseline of variables (*N* = 38)

Variables		BBSI (*n* = 20)	Control (*n* = 18)	*p*
		Mean ± SD or n (%)		
Age (yr)		80.05 ± 4.21	81.06 ± 4.32	.472
Education level	<Elementary school	17 (80.0)	15 (83.4)	.383
	≥ Middle school	3 (15.0)	3 (16.6)	
Spouse	Yes	3 (15.0)	3 (16.7)	.616^†^
	No	17 (85.0)	15 (83.3)	
Monthly income	< 100	20 (100.0)	17 (95.0)	.474^†^
(10,000 won)	≥ 100	0 (0.0)	1 (5.0)	
Parity (number)		3.80 ± 1.58	3.17 ± 1.92	.272
Caffeine intake (cup/day)	< 4	10 (50.0)	11 (61.1)	.492
	≥ 4	10 (50.0)	7 (38.9)	
Water intake (cc/day)	< 1000	12 (60.0)	15 (83.3)	.113^†^
	≥ 1000	8 (40.0)	3 (16.7)	
Regular exercise (/week)	< 3	17 (85.0)	15 (83.3)	.263^†^
	≥ 3	3 (15.0)	3 (16.7)	
BMI (kg/m^2^)		23.95 ± 3.96	24.13 ± 2.94	.882
OAB duration (yr)		2.35 ± 2.38	1.58 ± 1.29	.225^§^
HRV				
SDNN (ms)		29.79 ± 29.13	25.54 ± 19.48	.826^§^
RMSSD (ms)		37.02 ± 35.56	28.97 ± 23.93	.682^§^
TP (ms^2^)		2893.95 ± 6692.99	1303.61 ± 3158.63	.748^§^
LF (ms^2^)		1429.35 ± 3794.62	735.39 ± 1657.95	.815^§^
HF (ms^2^)		856.20 ± 1579.64	638.89 ± 1562.95	501^§^
LF/HF ratio		2.59 ± 2.23	2.81 ± 2.13	.520^§^
Catecholamines				
E (pg/mL)		42.31 ± 13.58	50.56 ± 18.73	.126^§^
NE (pg/mL)		472.84 ± 198.28	511.77 ± 195.55	.520^§^
OAB symptom severity		42.81 ± 10.59	38.54 ± 11.83	.248^§^
Sleep				
SE (%)		86.89 ± 4.93	87.77 ± 5.39	.619^§^
WASO (min)		69.20 ± 27.57	56.27 ± 22.94	.128^§^
Number of awakenings		10.85 ± 4.16	10.14 ± 4.80	.609^§^
Number of awakenings in 3 h after sleep onset		4.48 ± 1.74	4.09 ± 1.81	.702^§^

†Fisher’s exact test; § Mann-Whitney test; *BBSI* Biofeedback-based sleep improvement program, *OAB* Overactive bladder syndrome, *SDNN* Standard deviation of all normal to normal R-R intervals, *RMSSD* Root mean square differences of successive R-R intervals, *TP* Total power, *LF* Low frequency, *HF* High frequency, *Epi* Epinephrine, *NE* Norepinephrine, *SE* Sleep efficiency, *WASO* Wake after sleep onset

The effects of the 12-week BBSI program on the HRV parameters and plasma catecholamine levels are summarized in Table 2. Significant decreases were observed in RMSSD (37.02 ± 35.56 vs. 51.93 ± 49.61, *p* = 0.025), LF/HF ratio (2.59 ± 2.23 vs. 1.03 ± 0.27, *p* = 0.006), and epinephrine levels (42.31 ± 13.58 vs. 34.69 ± 12.83, *p* = 0.039) in the BBSI group after the intervention, whereas SDNN, TP, LF, and HF did not differ after the intervention. All the parameters related to HRV and plasma catecholamine levels in the control group did not change in the control group after the 12-week period.

**Table 2:** Effects of the BBSI program on the levels of heart rate variability parameters and catecholamines (*N* = 38)

Variables	BBSI (*n* = 20)			Control (*n* = 18)		
	Pre	Post		Pre	Post	
	Mean ± SD	Mean ± SD	*p*	Mean ± SD	Mean ± SD	p^*^
SDNN (ms)	29.79 ± 29.13	47.47 ± 37.51	.113	25.54 ± 19.48	21.63 ± 11.50	.420
RMSSD (ms)	37.02 ± 35.56	51.93 ± 49.61	.025	28.97 ± 23.93	29.39 ± 22.45	.758
TP (ms^2^)	2893.95 ± 6692.99	2036.60 ± 4422.05	.550	1303.61 ± 3158.63	2302.22 ± 5030.27	.983
LF (ms^2^)	1429.35 ± 3794.62	804.85 ± 2132.54	.455	735.39 ± 1657.95	1074.83 ± 2463.03	.433
HF (ms^2^)	856.20 ± 1579.64	1147.25 ± 2163.45	.709	638.89 ± 1562.95	844.33 ± 1893.83	.647
LF/HF ratio	2.59 ± 2.23	1.03 ± 0.27	.006	2.81 ± 2.13	2.42 ± 1.30	.446
Epi (pg/mL)	42.31 ± 13.58	34.69 ± 12.83	.039	50.56 ± 18.73	59.20 ± 23.59	.076
NE (pg/mL)	472.84 ± 198.28	434.91 ± 180.38	.218	511.77 ± 195.55	457.87 ± 210.90	.129

*BBSI* Biofeedback-based sleep improvement program, *OAB* Overactive bladder syndrome; *p*: difference from pre-test to post-test in BBSI groups p^*^: difference from pre-test to post-test in control groups, *SDNN* Standard deviation of normal to normal R-R intervals, *RMSSD* Root mean square differences of successive R-R intervals, *TP* Total power, *LF* Low frequency, *HF* High frequency, *E* Epinephrine, *NE* Norepinephrine

The effects of the 12-week BBSI intervention on OAB symptoms measured by OAQ-q are summarized in Table 3. At baseline, no significant difference was found in the frequency, urgency, nocturia, or urgency incontinence between the patients and controls (Table 1). After the 12-week intervention, the BBSI group demonstrated a significant improvement in the urgency score (8.5 ± 1.5 vs. 7.5 ± 0.82, *p* = 0.004), nocturia (9.35 ± 2.08 vs. 6.85 ± 1.42, *p* < 0.001), and total score (48.81 ± 10.59 vs. 33.96 ± 7.31, *p* < 0.001).

**Table 3:** Effects of BBSI program on the severity of OAB symptoms, as measured by OAB-q (*N* = 38)

Variables	BBSI (*n* = 20)			Control (*n* = 18)		
	Pre	Post		Pre	Post	
	Mean ± SD	Mean ± SD	*p*	Mean ± SD	Mean ± SD	*p* ^*^
Frequency	2.00 ± 0.79	1.95 ± 0.51	.782	2.22 ± 0.73	1.94 ± 0.54	.132
Urgency	8.50 ± 1.50	7.50 ± 0.82	.004	7.50 ± 1.00	7.39 ± 1.58	.756
Nocturia	9.35 ± 2.08	6.85 ± 1.42	<.001	8.44 ± 2.06	8.39 ± 1.29	.979
Urgency incontinence	6.70 ± 2.11	6.00 ± 1.84	.100	6.33 ± 3.12	5.33 ± 2.33	.055
Total	42.81 ± 10.59	33.96 ± 7.31	<.001	38.54 ± 11.83	35.53 ± 9.44	.214

*BBSI* Biofeedback-based sleep improvement program, *OAB* Overactive bladder syndrome, *OAB-q* Overactive bladder questionnaire; *p*: difference from pre-test to post-test in BBSI groups; p^*^: difference from pre-test to post-test in control groups

Table 4 shows that, regarding sleep patterns, in the BBSI group, we observed a significant difference in SE, WASO, number of awakenings, and number of awakenings within three hours after sleep onset (*p* = 0.004, *p* = 0.001, *p* = 0.001, and *p* = 0.048, respectively).

**Table 4:** Effects of the BBSI program on the patterns of sleep parameters (*N* = 38)

Variables	BBSI (*n* = 20)			Control (*n* = 18)		
	Pre	Post		Pre	Post	
	Mean ± SD	Mean ± SD	*p*	Mean ± SD	Mean ± SD	*p* ^*^
SE (%)	86.89 ± 4.93	90.54 ± 4.36	.004	87.77 ± 5.39	86.71 ± 6.07	.717
WASO (min)	69.20 ± 27.57	37.28 ± 21.16	.001	56.27 ± 22.94	59.48 ± 22.87	.281
Number of awakenings	10.85 ± 4.16	7.12 ± 2.89	.001	10.14 ± 4.80	10.94 ± 4.21	.236
Number of awakenings in 3 h after sleep onset	4.48 ± 1.74	3.44 ± 1.74	.048	4.09 ± 1.81	4.65 ± 2.29	.256

*BBSI* Biofeedback-based sleep improvement program, *OAB* Overactive bladder syndrome; *p*: difference from pre-test to post-test in BBSI groups; p^*^: difference from pre-test to post-test in control groups; *SE* Sleep efficiency, *WASO* Wake after sleep onset

## Discussion

In this study, we aimed to develop, apply, and analyze the efficacy of a BBSI program in elderly women with OAB who were assumed to have an overall ANS imbalance due to excessive sympathetic activity. The program included biofeedback training using HRV, lifestyle modifications, physical activity, depression management, sleep hygiene, and PFM exercises. After the 12-week program, RMSSD—a key indicator of HRV—had increased significantly, while the LF/HF ratio and epinephrine secretion decreased significantly. Furthermore, OAB symptoms had alleviated. These results improved sleep by decreasing WASO, number of awakenings during sleep, number of awakenings within 3 h of sleep onset, and increasing sleep efficiency (SE).

Notable results of this study were that post-intervention RMSSD in the experimental group increased significantly, whereas the post-intervention LF/HF ratio decreased significantly. No differences were observed in these indicators in the control group after completion of the study. Biofeedback using HRV effectively treated the psychological issues, such as depression, panic, and insomnia through ANS balance [17]. The ANS also demonstrated other benefits, such as decreased pulse, increased body temperature, and improved blood circulation [37].

According to previous studies in females with OAB, the LF/HF ratio is significantly increased when the bladder is full [38, 39]. Hsiao et al. emphasized that the sympathetic nervous system is relatively overactive in OAB because the LF/HF ratio is higher in women with OAB than that in healthy adults, even when the bladder is empty [40]. Similarly, Choi et al. [41] reported that HF—a frequency domain indicator of RMSSD and parasympathetic nerve activity—was decreased in women with OAB compared to healthy adults. RMSSD increased and LF/HF ratio decreased after the intervention in this study—a finding that was consistent with ANS changes that may help alleviate OAB symptoms. Furthermore, Hubeaux et al. suggested that ANS dysfunction due to sympathetic predominance may be implied in the generation of abnormal bladder sensations, such as urgency [38, 39]. In contrast, increased parasympathetic nerve activity has been reported as a cause of OAB [7, 42] or nocturia [43]. There are differences in the participants and data collection methods in these studies; therefore, direct comparison is difficult. However, our biofeedback protocol was designed to activate the parasympathetic nerves while keeping the LF/HF ratio at an appropriate value during biofeedback training, which may have been an important factor in alleviating OAB symptoms. Considering the results of previous studies, the results of this study confirm the possibility of ANS imbalance due to sympathetic dominance as the cause of OAB. ANS changes associated with senescence involve increase and decrease in the activities of the sympathetic and parasympathetic nervous systems, respectively [10, 44]. Such changes lead to sleep disturbances, including frequent awakening and reduced rapid eye movement (REM) sleep [44]. In one study, RMSSD was reported to be lower in elderly individuals than that in younger individuals, and this finding was more common in females [45]. Therefore, increased RMSSD may induce deep sleep and stabilize sleep structure. Further, serum epinephrine was significantly reduced after 12 weeks in the BBSI group. The LF/HF ratio is well-known to better reflect sympathetic nervous system balance than any other single indicator [30], whereas the circulating epinephrine level is directly related to sympathetic activity. Therefore, serum epinephrine level is a powerful tool for evaluating the program efficacy.

Urinary symptom severity was significantly decreased in the experimental group, while no differences were noted in the control group. Symptoms of OAB are directly affected by changes in sleep structure due to aging, such as decreased REM and slow wave sleep [5]. Active interventions for these direct factors play an important role in improving sleep in elderly individuals [46]. The primary intervention for alleviating urinary symptoms is behavioral therapy, especially in elderly patients. This treatment is safer and has fewer side effects than drug therapy or surgery [47, 48] and has better subjective assessment of symptoms than drug therapy [49].

Few previous studies have focused on ANS as a mediator in the relief of OAB symptoms. Cho et al. [50] attempted to alleviate OAB symptoms by inducing ANS changes using Tai-chi. No change in ANS activity was observed but urinary symptoms were alleviated without improvement in nocturia [50]. However, in the present study, nocturia and urgency were improved—but not frequency and urgency incontinence. We believe that this difference in the outcome was due to the focus of the intervention. In OAB patients—especially those with urgency incontinence—it is important to acquire skills to respond to urgency situations, such as PFM contraction and relaxation techniques [48]. However, the intervention program of this study was composed mainly of factors that reduce nocturia by emphasizing the practice of sleep hygiene—including water and caffeine restriction—urination before bedtime, and performing appropriate exercise to reduce awakening. These results indicate that urinary symptoms are related to several factors and that a customized intervention for each symptom is necessary for symptomatic relief [47].

In this study, SE increased, while wakefulness after sleep onset, number of awakenings during sleep, and number of awakenings within 3 h after sleep onset decreased, and overall sleep improved in the experimental group after the program. In elderly women with OAB, SE reduced and WASO increased significantly due to decreased urinary symptoms and exacerbated changes that occur during the normal aging process [5, 51]. Furthermore, since sympathetic nervous activity may affect REM sleep and induce nocturia [52], these results suggest that increasing RMSSD and decreasing the LF/HF ratio improves sleep in elderly women with OAB. Particularly, lower sleep quality due to nocturia is a problem due to which patients with OAB are the most inconvenienced [53]. Decreased wakefulness after sleep onset is associated with reduced nocturnal enuresis. Additionally, the frequency of awakening due to urination in the initial stages of sleep is a key parameter for clinically assessing the quality of sleep and life [54]. The frequency of awakening during sleep is known to increase with age and this frequency is important in terms of sleep quality in elderly individuals with OAB because it can be difficult to return to sleep after awakening [55]. The elderly demonstrate slow sleep—which is deep sleep for 3 h after sleep onset—during which arousal has the greatest effect on daytime drowsiness and disruption of daily activities [56]. The decrease in awakening frequency seems to positively affect sleep quality by increasing sleep continuity.

This study introduced a method to effectively treat sleep disorders using a program that addresses the physiological issues to alter ANS imbalances. A strong point of the program is that it addresses the issue integrally by approaching sleep disruption in view of the characteristics of OAB. Additionally, as an early study of sleep problems in patients with OAB, considerable results were obtained that confirm that ANS stabilization is effective in improving OAB and sleep. Based on the results of this study, it also may be possible to apply ANS stabilization to OAB patients more easily in daily life, such as relaxation and meditation therapy [57].

Despite the strengths, the study has some limitations. First, the study has the potential for bias due to the use of a non-randomized controlled trial design and no blinding between the researchers and participants. Second, HRV measurements—reflecting ANS—were evaluated during daytime stability and do not reflect changes in sleep and bladder activity at other times of the day. Third, the findings should be generalized with caution as there is the possibility of interference from external variables and the fact that only two senior welfare centers in Korea were included. Finally, it is difficult for the participants to apply the BBSI program’s protocol to their daily life on their own. Further studies with larger samples with randomized control trial designs and direct ANS activity measurements are needed to evaluate the precise relationships between ANS activity, bladder activity, and sleep patterns. Additionally, simplifying the elements of the program and making it easier to apply in everyday life will help the participants manage the disorders on their own.

## Conclusions

This study tested a BBSI program in elderly women with OAB in a coordinated manner and demonstrated that the program could improve ANS balance, alleviate urinary symptoms, and improve sleep. The results of this intervention study demonstrate that it is meaningful to consider physiological factors in sleep interventions. Therefore, the program is expected to positively affect the quality of sleep and life in elderly women with OAB.

## Supplementary information

**Additional file 1.** Biofeedback-based sleep improvement program

## Data Availability

The datasets used and/or analyzed in the current study are available from the corresponding author upon reasonable request, in compliance with the policies and procedures of the Bisa Research Grant of Keimyung University for data sharing.

## Abbreviations

ANS: Autonomic nervous system
BBSI: Biofeedback-Based Sleep Improvement
Epi: Epinephrine
HF: High frequency
HRV: Heart rate variability
LF: Low frequency
MMSE: Mini-Mental State Examination
NE: Norepinephrine
OAB: Overactive bladder syndrome
OABSS: Overactive bladder symptom scores
PFM: Pelvic floor muscle
PSQI: Pittsburgh Sleep Quality Index
RMSSD: Square root of the mean squared differences of successive N-N intervals
SDNN: Standard deviation of the N-N interval
SE: Sleep efficiency
TP: Total power
WASO: Wake after sleep onset
