# Supplementary material for: Effects of biofeedback-based sleep improvement program on urinary symptoms and sleep patterns of elderly Korean women with overactive bladder syndrome
Source: BMC Urol. 2019 Nov 8;19:109. doi: 10.1186/s12894-019-0540-y (PMC6839159; doi:10.1186/s12894-019-0540-y)
Supplement: Supplementary file 1 — Additional file 1. Biofeedback-based sleep improvement program [file 12894_2019_540_MOESM1_ESM.docx]

Additional File 1. Biofeedback-based sleep improvement program

| Week | Theme | Method | Intervention | | Duration |
| --- | --- | --- | --- | --- | --- |
| 1 | Beginning | Group | · Introduce the program  · Introduce oneself and motive for participation  · Initial assessment of overactive bladder (OAB) symptoms and sleep patterns  · Pre-test | | 40 min. |
|  |  | Individual | Biofeedback | · Introduce biofeedback  · Initial assessment of respiratory rate | 20 min. |
|  |  |  | Telephone coaching | · Encourage and support participant to complete the 12-week program | 10 min./2 sessions |
| 2 | Preparing to take a good sleep | Group | · OAB and sleep  · Identify factors that hinder good sleep and effort taken to take a good sleep  · Keep a sleep diary and bladder diary  · Deep breathing | | 40 min. |
|  |  | Individual | Biofeedback | · Abdominal breathing and pursed-lip breathing education  · Breathing training | 20 min. |
|  |  |  | Telephone coaching | · Encourage participant to practice things learned in week 2 | 10 min./2 sessions |
| 3 | Practicing  sleep hygiene Ⅰ | Group | · Check sleep diary and bladder diary  · Explain about the concept of sleep hygiene  · Session 1 of sleep hygiene education  · Provide and encourage participant to fill out a weekly sleep hygiene checklist  · Deep breathing | | 40 min. |
|  |  | Individual | Biofeedback | · Abdominal breathing and pursed-lip breathing education  · Breathing training | 20 min. |
|  |  |  | Telephone coaching | · Encourage participant to practice things learned in week 3 | 10 min./2 sessions |
| 4 | Practicing  sleep hygiene Ⅰ | Group | · Check practice of week 3 sleep hygiene contents and share about difficulties  · Encourage participant to practice things learned in session 1 of sleep hygiene education  · Check weekly sleep hygiene checklist  · Deep breathing | | 40 min. |
|  |  | Individual | Biofeedback | · Abdominal breathing and pursed-lip breathing education  · Breathing training | 20 min. |
|  |  |  | Telephone coaching | · Identify difficulties with practicing contents of week 4 education | 10 min./2 sessions |

(Continued)

Table 1. (Continued)

| Week | Theme | Method | Intervention | | Duration |
| --- | --- | --- | --- | --- | --- |
| 5 | Practicing  sleep hygiene  Ⅱ | Group | · Session 2 of sleep hygiene education  · Provide and encourage participant to fill out the weekly sleep hygiene checklist  · Deep breathing | | 40 min. |
|  |  | Individual | Biofeedback | · Abdominal breathing and pursed-lip breathing education  · Breathing training | 20 min. |
|  |  |  | Telephone coaching | · Identify difficulties with practicing contents of week 5 education | 10 min./2 sessions |
| 6 | Practicing  sleep hygiene  Ⅱ | Group | · Check practice of week 5 sleep hygiene contents and share about difficulties  · Encourage participant to practice things learned in session 2 of sleep hygiene education  · Check weekly sleep hygiene checklist  · Deep breathing | | 40 min. |
|  |  | Individual | Biofeedback | · Abdominal breathing and pursed-lip breathing education  · Breathing training | 20 min. |
|  |  |  | Telephone coaching | · Identify difficulties with practicing contents of week 6 education | 10 min./2 sessions |
| 7 | Interim evaluation | Group | · Check weekly sleep hygiene checklist  · Award students for excellent use of the sleep hygiene checklist and awardees share about their experiences  · Encourage adherence to sleep hygiene  · Deep breathing | | 40 min. |
|  |  | Individual | Biofeedback | · Abdominal breathing and pursed-lip breathing education  · Breathing training | 20 min. |
|  |  |  | Telephone coaching | · Provide support to help participants to practice things learned in week 7 | 10 min./2 sessions |
| 8 | A good sleep begins with your daily life | Group | · Educate about the need for lifestyle modification  · Caffeine and alcohol restriction and BMI management  · Deep breathing | | 40 min. |
|  |  | Individual | Biofeedback | · Abdominal breathing and pursed-lip breathing education  · Breathing training | 20 min. |
|  |  |  | Telephone coaching | · Provide support to help participants to practice things learned in week 8 | 10 min./2 sessions |

(Continued)

Table 1. (Continued)

| Week | Theme | Method | Intervention | | Duration |
| --- | --- | --- | --- | --- | --- |
| 9 | A healthier sleep  Ⅰ | Group | · Physical activity and sleep in OAB  · Setting the goal for physical activity: Provide a pedometer  · Introduce pelvic floor muscle training  · Practice pelvic floor muscle training  · Deep breathing | | 40 min. |
|  |  | Individual | Biofeedback | · Abdominal breathing and pursed-lip breathing education  · Breathing training | 20 min. |
|  |  |  | Telephone coaching | · Provide support to help participants to practice things learned in week 9 | 10 min./2 sessions |
| 10 | A healthier sleep  Ⅱ | Group | · Check on the status of goal achievement  · Set new goals and share experiences among groups  · Encourage pelvic floor muscle training  · Compliment excellent participants | | 40 min. |
|  |  | Individual | Biofeedback | · Abdominal breathing and pursed-lip breathing education  · Breathing training | 20 min. |
|  |  |  | Telephone coaching | · Provide support to help participants to practice things learned in week 10 | 10 min./2 sessions |
| 11 | A healthier sleep  Ⅲ | Group | · Keep a sleep diary and bladder diary  · Increase amount of physical activity  · Encourage pelvic floor muscle training  · Deep breathing | | 40 min. |
|  |  | Individual | Biofeedback | · Abdominal breathing and pursed-lip breathing education  · Breathing training | 20 min. |
|  |  |  | Telephone coaching | · Provide support to help participants to practice things learned in week 11 | 10 min./2 sessions |
| 12 | A healthier tomorrow | Group | · Concluding the program  · Participants share their experience with the program and about themselves  · Post-test  · Completion ceremony | | 40 min. |
|  |  | Individual | Biofeedback | · Abdominal breathing and pursed-lip breathing education  · Breathing training | 20 min. |
|  |  |  | Telephone coaching | · Encourage participant to continue their effort to enhance sleep | 10 min./2 sessions |
